# “Ọmụgwọ” As Unpaid Labor? The Perceptions of Postpartum Caregiving Among Older Grandmothers in Southeast Nigeria

**DOI:** 10.1093/geroni/igad069

**Published:** 2023-07-13

**Authors:** Anthony Obinna Iwuagwu

**Affiliations:** Department of Social Work, University of Nigeria, Nsukka, Nigeria

**Keywords:** Africa, Aging, Older adults, Women

## Abstract

**Background and Objectives:**

In Nigeria and many Sub-Saharan African countries where the majority are poor and cannot afford formal postpartum care, nursing mothers rely primarily on their mothers or older female relatives for postpartum care. Despite their invaluable contributions, such grandmothers often operate in a reality of inadequate social and institutional support. Yet, little is known about how women perceive this form of traditional care in Sub-Saharan Africa. This study explored the views of older grandmothers in Southeast Nigeria about postpartum caregiving, called “Ọmụgwọ.”

**Research Design and Methods:**

Using a qualitative descriptive inquiry, the author employed criterion-purposive sampling and snowballing to recruit 17 older grandmothers who participated in the interviews, and data were analyzed in themes.

**Results:**

Three themes and eight subthemes emerged. The themes cover perceptions supporting the continuity of Ọmụgwọ practice irrespective, the influence of culture on Ọmụgwọ practice, and the indirect rewards of the practice.

**Discussion and Implications:**

The findings of this study could potentially influence postpartum caregiving policies for female older adults in Sub-Saharan Africa and further advance the quality of informal care during postpartum periods in Africa.

**Translational Significance:** The findings of this study could potentially influence postpartum caregiving policies for female older adults in Sub-Saharan Africa and further advance the quality of informal care during postpartum periods in Africa. Gerontological social workers among other clinicians could leverage these experiences to improve the health and mental well-being of these populations in the region. For instance, by building on their cultural views of Ọmụgwọ, gerontological social workers could help facilitate invites and travels for Ọmụgwọ as this could potentially reduce social loneliness among their patients.

Ọmụgwọ—an aspect of unpaid care labor is an important part of the economy as well as an essential contributor to the well-being of individuals, families, and societies (Stiglitz et al., 2007). Despite its relevance to family well-being, Ọmụgwọ like other forms of unpaid caregiving is frequently left off research and policy agendas (Ferrant et al., 2014). Ọmụgwọ is akin to postpartum caregiving—defined as a situation where women (usually maternal grandmothers of the newborn) are invited to care for and mentor nursing mothers about childcare without an established remuneration. This can occur for a firstborn child or subsequent births.

Globally, three quarters of neonatal deaths occur in the first week of life, with about half of those occurring at home (Bhutta et al., 2014; Lawn et al., 2005). In low-resource countries, between 20% and 44% of maternal deaths are estimated to occur in the postpartum period (Tessema et al., 2017). Studies have shown that adequate care provision during the postpartum period could save more than half of all neonatal fatalities and maternal emergencies (Tiruneh et al., 2019; Zephyrin et al., 2019). The World Health Organization (WHO) therefore recommends postnatal care for nursing mothers and their babies upon delivery, preferably by trained professionals (2015). However, in most low- and middle-income countries, such as Nigeria, where professional care is expensive, experienced family members, usually grandmothers of the newborn, provide unpaid postpartum care (Bergsjø, 1993). Ojembe and Kalu (2018) aver that the concept of reciprocity and family care characterize caregiving in Africa—where providing unpaid care for a nursing mother and her baby is cultural and revered. Gender and age of grandparent overlaps and being female compounds the experience of financial hardship among persons aged 60 years and older compared to their male counterpart (Feldman & Radermacher, 2016). According to Australian Human Rights Commission (2018), women are less likely than men to be able to amass and maintain wealth throughout the course of their lives. Women’s income and work cycle differ from men’s; they frequently enter and exit the workforce due to care responsibilities, work in lower-paying jobs, and experience difficulty finding new jobs (Australian Human Rights Commission, 2018). In Africa, studies have also reported that women generally are at higher risk of poverty and financial hardship (Kaka, 2013; Milazzo & van de Walle, 2017; Mwakalila, 2022).

As life expectancy increases globally, the number of grandparents will increase. For instance, of the 7.6 billion individuals worldwide, 1.4 billion, or 18%, are grandparents (Chamie, 2018), the majority of whom are women (Shmerling, 2020). According to the literature, it is common practice for nursing mothers to be cared for by their mothers or mothers-in-law in many cultures, particularly in Sub-Saharan Africa (Bergsjø, 1993; Chukwu & Ume, 2020). These grandmothers handle household duties as well as general caregiving for the nursing mother and the newborn, allowing the nursing mother enough time to rest and recover (Igbokwe & Ahurumaraeze 2019; Umunna 2012). For instance, in parts of Tanzania and Kenya, the women (Maasai women) are cared for and encouraged to focus their attention on their nutrition, recovery, and breastfeeding alone (Bergsjø, 1993). Whereas the grandmother of the newborn takes responsibility for house chores, cares for the nursing mother and the newborn, and mentors new mothers in the role of childcare (Chukwu & Ume, 2020). In some parts of Nigeria, the nursing mothers and the newborn are encouraged to remain in a room for adequate rest, recovery, and weight gain (Kelly, 1967), whereas the grandmother prepares local delicacies to facilitate recovery for the nursing mother (Ujumadu, 2018). In Southeast Nigeria, the ceremony that surrounds this period of care is referred to as the “Ọmụgwọ ritual” (care after childbirth) (Ekweariri, 2020). Ọmụgwọ is prioritized for a first-birth experience due to the inexperience of the new mother and the need to be cared for and mentored. However, the practice often continues to other births as an entitlement to grandmothers and sometimes to encourage relaxation of grandmothers including those who are very old, sick, and cannot engage in the actual care. Ohaja and Anyim (2021) noted that the duration of Ọmụgwọ differs and ranges from a minimum of 7 weeks to 6 months of unpaid caregiving.

Unpaid care work is often perceived as low value and is invisible in mainstream economics, underpinned by entrenched patriarchal institutions and national accounting systems that fail to factor in women’s total contributions. According to the International Labour Organization (ILO), if care labor was valued equally to other jobs, it would account for a tenth of global economic production (ILO, 2018). Women’s experience of unpaid domestic work and care, and the drudgery associated with these activities, vary greatly, not only between high- and lower-income countries but also among different income groups within countries (United Nations Women, 2020). For instance, in low- and middle-income countries such as Nigeria, most grandparents of working age who are willing and able to work are not able to find work (Sasu, 2022). The same cannot be said of their peers in high-income economies with higher employment rates (ILO, 2020). As a result, the level of income may affect perceptions and participation in postpartum caregiving across nations. Further, most unpaid caregivers especially in low- and middle-income countries such as Nigeria regard unpaid caregiving as a privilege to spend time experiencing the emotional and relational components of direct care for children, grandchildren, parents, or other care receivers (ILO, 2018). However, in more industrialized nations unpaid domestic and care work is associated with greater mental health burden and negative effects on quality of life (Pinquart & Sörensen, 2003; Vitaliano et al., 2003).

Studies have investigated unpaid labor, with women at the center of the investigation (Seedat & Rondon, 2021; Singh & Pattanaik, 2020). Others have explored varied areas of caregiving including the burden of care, conflicts in caregiving, and challenges of caregiving among women (DiGirolamo & de Snyder, 2008; Power, 2020; Rajanala et al., 2020). However, these studies focus largely on younger and middle-aged caregivers (paid and unpaid). In Nigeria, although much is understood about informal/unpaid caregiving and that women make up most of the informal caregivers (Agbawodikeizu et al., 2021; Ebimgbo et al., 2019; Okoye, 2012), there is a rareness of literature on the experiences of grandmothers as caregivers involved in Ọmụgwọ. It is important to shine the investigative light of research on these populations and care period, given the intersectionality of their age, gender, and experience of Ọmụgwọ which could potentially affect them in areas including health, income, gender equality, and empowerment (Iwuagwu, Ngwu, et al., 2022; Kelly et al., 2019). This study, therefore, aims to contribute to the literature on caregiving in Nigeria by exploring the perceptions of older grandmothers about Ọmụgwọ—in relation to unpaid labor. Findings of this study, if adopted, could potentially influence caregiving policies for older adults in Sub-Saharan Africa and further advance quality informal care during Ọmụgwọ in Africa, where the issue is prevalent.

## Materials and Methods

### Study Design

The sampling, data collecting, and analysis for this study were guided by the qualitative description research design (Sandelowski, 2000). Qualitative description design is most appropriate for this study compared to other designs because it does not aim to generalize but to explore the depth and details of a phenomenon through the experiences of the participants and how they make meaning of those experiences (Sandelowski, 2000). Utilizing this approach, the researcher was able to describe the views of grandmothers in Nigeria about postpartum care as unpaid labor. Data were gathered from older adults using semi-structured interviews. This study received ethical approval from the Health Research Ethics Committee, University of Nigeria Teaching Hospital (NHREC/05/01/2008B-FWA00002458-1RB00002323), having followed the guidelines of the Helsinki Declaration on research ethics (World Medical Association, 2013). Only participants who gave written or verbal consent were allowed to take part in the study. The Consolidated Criteria for Reporting Qualitative Studies were followed (Tong et al., 2007).

### Study Setting, Sampling, and Recruitment

The researcher conducted this study in Imo State, Nigeria. Imo State is one of the five Southeast states in Nigeria and is predominantly of Igbo extraction. Imo State has an estimated population of 6,347,078 (Igwenagu, 2021) and has one of the highest populations of older adults in the country (Imo State Government, 2012). Participants were recruited through study announcements, flier distribution, word of mouth, and directly reaching out in social gatherings, such as places of worship, women’s fellowship, and women’s annual meetings in Southeast Nigeria popularly called “August meeting.” Those who were interested in the study contacted the researcher directly and/or through telephone contact provided. Following the tenets of the criterion-based purposive sampling in selecting participants, the researcher included only older adults who are (1) 60 years or older and a grandmother, (2) able to consent and communicate in English, Igbo, or Nigerian pidgin, (3) self-identify as a person with at least 2 months of postpartum caregiving experience, and (4) reside in Imo State, Nigeria. Two months aligns with the lower range (7 weeks) of the duration of Ọmụgwọ in Nigeria (Ohaja & Anyim, 2021). Using the snowball recruitment strategy, participants were requested to suggest and provide contact information of others who met the inclusion criteria for participation. The researcher continued to contact participants through this method until data saturation, defined as when no new information emerges during interviewing, was reached (Braun & Clarke, 2022). As a result, data collection stopped after 17 interviews when data saturation was achieved as data became repetitive (see Table 1).

**Table 1.** Demographic Information of Participants

Name	Age (years)	Highest level of education	Locality	Professional status
Mrs. Nkem	61	No formal education	Rural	Unemployed
Mrs. Ngo	60	No formal education	Rural	Unemployed
Mrs. Amaka	63	WASSCE	Rural	Unemployed
Mrs. Joy	60	PhD	Urban	Civil servant
Mrs. Onyi	61	BSs	Urban	Trader
Mrs. Victoria	69	BSc	Urban	Retiree
Mrs. Uwaoma	71	FLSC	Rural	Unemployed
Mrs. Okeke	65	FSLC	Rural	Unemployed
Mrs. Omeke	70	FSLC	Urban	Unemployed
Mrs. Ihuoma	61	BSs	Rural	Unemployed
Mrs. Chioma	72	WASSCE	Rural	Retiree
Mrs. Maggi	60	WASSCE	Rural	Trader
Mrs. Chichi	64	MSc	Rural	Retiree
Mrs. Chinenye	64	MSc	Urban	Civil servant
Mrs. Akudo	60	WASSCE	Rural	Unemployed
Mrs. Chukwu	61	FSLC	Rural	Unemployed
Mrs. Ugodi	60	BSc	Urban	Unemployed

*Notes*: BSc = Bachelor of Science degree; FSLC = First School Leaving Certificate (certificate issued after completing 6 years of primary education); MSc = Master of Science degree; PhD = Doctor of Philosophy/Doctorate degree; WASSCE = West African Senior Secondary Certificate Examination (certificate issued after completing 6 years of secondary school).

### Demographic Characteristics of Participants

All the participants are female, aged between 60 and 72 years, with a mean (SD) age of 66 years. The majority (64.7%) of the participants had attained a secondary level (A-level) education or higher and they (64.7%) live in rural areas. Majority (58.8%) of the participants were unemployed, whereas others were retirees, civil servants, and traders (classified as economically engaged).

### Data Collection

Data were collected between July and September 2021. Participants were invited to a single, in-depth, semi-structured interview. Interviews were done by the researcher, who speaks English, Igbo, and Nigerian pidgin fluently. The participants received a participant information sheet (PIS), which outlined the research aims, risks, benefits of participation, and the need for recording. The PIS contents were read to those who had difficulty reading in English. The possibility to withdraw from the study at any moment and other ethical issues like confidentiality and anonymity were also discussed with the participants. Participants provided informed consent to the researcher to participate in the study and agreed to record their opinions. Thirty women indicated an interest in the research but only 21 met the inclusion criteria. However, three dropped out because of scheduling issues, and halfway into an interview, a participant withdrew their consent to participate for personal reasons. Interviews were conducted using a semi-structured interview guide with probing questions as an instrument for data collection. Examples of the questions the researcher asked participants include: (a) What are your views about Ọmụgwọ in relation to unpaid labor?; (b) What are your personal experiences of Ọmụgwọ?; and (c) What factors influence your perception and practice of Ọmụgwọ in Southeast Nigeria? Participants were probed based on their responses during the interviews. Participants were asked to provide examples to support any key arguments or statements they made. Although the participants were provided with the option to speak either Igbo, English, or Nigerian pidgin, they all chose English and Igbo. Only five interviews were conducted in Igbo, whereas the rest spoke English. The researcher conducted a pilot interview with three older adults who fit into the study criteria to determine the appropriateness of the questions to ask and the appropriate length of time to allow for each interview (piloted data were not included in this study). Each interview lasted for 50–60 min.

### Data Analysis

Data analysis was done simultaneously with data gathering, which allowed the researcher to achieve data saturation (Saunders et al., 2018). For example, recordings were coded as they are gotten a participant pending the date and time for the next interview. By implication, this will allow the researcher to notice data saturation and to inform when to stop interviewing. Recorded interviews were transcribed verbatim in English, while the five interviews in Igbo were translated to English by two expert translators from the Department of Igbo Linguistics of the Author’s University, and thereafter, transcribed using the parallel transcription framework described by Nikander (2008). The transcripts were then compared against the recordings of the interviews and field notes to make sure they accurately captured the original meanings and concepts and to assure the accuracy of the data (Kalof et al., 2008). Afterward, the NVivo 12 qualitative analysis program was used to code the data. Instead of using preexisting codes or coding schemes designed in advance, an inductive approach (A method that allowed codes to come to the researcher’s mind while reading the transcripts and reviewing the literature) was used to code the transcribed data. The data were analyzed using the thematic approach, which entails identifying, analyzing, and reporting patterns of meaning in data (Braun & Clarke, 2006; Ritchie et al., 2014). Firstly, the units of analysis were defined—sentences on Ọmụgwọ experiences from the grandmothers’ perspective. Secondly, one coder read the transcripts line by line to generate initial codes, which were presented to a group of researchers (qualitative analysis experts) for feedback. Thirdly, a working draft coding book was developed, and different units of analysis were identified and applied as the author continued data collection and analysis. Although the author had a working draft coding book, the author continued to code line by line so that the original views of the respondents would not be lost (Braun & Clarke, 2006). See Supplementary Material for the coding sampling. The findings were written up, and topics that lacked sufficient evidence to back them were disregarded. Pseudonyms (fake names) were used to ensure the anonymity of participants in analyzing and reporting data. NVivo software was used to manage the qualitative analysis.

### Strategies to Ensure Trustworthiness

To ensure rigor, different strategies were employed (Lincoln & Guba, 1985). First, the researcher did a random participant-member checking based on availability. Seven participants were contacted at the end of the study analysis, and they all confirmed and validated the findings as a reflection of their views. To increase reflexivity, field notes (reflective and observation notes) were kept throughout the research process, which detailed the researcher’s “Subjective I”—the values, attitudes, and beliefs that a qualitative researcher holds, which could influence the research and study analysis (Kalu, 2019). For instance, the researcher is a Nigerian from the Igbo extraction, where Ọmụgwọ is practiced and known. Throughout the data collection and analysis, the author kept reflecting on how being Igbo could influence the data collection and analysis.

## Findings

Three major themes and eight subthemes were identified from the narratives of the respondents. The major themes reflect the perceptions supporting the continuity of Ọmụgwọ practice irrespective, the influence of culture on Ọmụgwọ practice, and the indirect rewards of the practice. Table 2 highlights the major themes and associated subthemes that emerged from the data analysis.

**Table 2.** Themes and Subthemes

Main themes	Subthemes
Positive outlook and continuity	• Relief for nursing mothers • Life rescue • Practice continuity irrespective
Cultural influences	• Care reciprocity and responsibility • Social status and self-esteem
Indirect care rewards/benefits for grandmothers	•Decreased loneliness •Tangible gifts •Tourism

### Theme 1: Positive Outlook and Continuity

Findings show that participants’ views supported the continuity of Ọmụgwọ practice irrespective. They hold a positive perception of Ọmụgwọ, and do not view it as unpaid labor, rather they believe it has many benefits for the health and well-being of the nursing mother and newborn, thus should continue. Specifically, they perceive Ọmụgwọ as having reduced caregiving stress for nursing mothers (their daughters) and reduced neonatal mortality and maternal mortality. Most of the participants were happy with the practice, are unconcerned about payments, and look forward to its continuity and their next Ọmụgwọ.

#### Relief for nursing mothers

Although participants highlighted the perceived impact of Ọmụgwọ practice in reducing stress for the nursing mother, most reflected on the negative effect of not having help after childbirth. Participants underscored that mothers who do not receive “Ọmụgwọ” practice are more prone to stress than those who receive it. The below quote highlights this:

I acknowledge the physical and mental stress I went through decades ago when I had my second child and had to care for myself and the newborn alone, due to my mother being late (dead) and my mother-in-law could not come for Ọmụgwọ as planned. It was a very hectic period for me, and I still wonder how I survived … without a doubt, nursing mothers who invite their mothers for Ọmụgwọ enjoy a stress-free postpartum period. (Mrs. Chioma, 72 years old)

… after just one week of postpartum care alone, she [My daughter] is complaining bitterly about her stress level and the need for me to come to Lagos for Ọmụgwọ as soon as possible. (Mrs. Ogodi, 60 years old)

#### Life rescue

It was noted that participants unanimously perceive Ọmụgwọ as having many health benefits as it has drastically reduced neonatal and maternal mortality among their children and grandchildren. Some of the participants believed that the experiences of grandmothers play an important role in preserving the life of the nursing mother and the child during Ọmụgwọ, whereas others compared the experiences of deaths between those who benefited from the Ọmụgwọ practice and those who did not. As a clear representation of the views and experiences of participants about the health benefits of Ọmụgwọ, the narratives of two respondents were presented thus:

Surely, Ọmụgwọ helps to reduce deaths among nursing mothers and newborns. Those who come for Ọmụgwọ are experienced in childcare and maternal care. They are often very knowledgeable about the medications and immunizations of children and the recovery process for nursing mothers. (Mrs. Joy, 60 years old)

… Personally, the only grandchild I have ever lost to death is the one whose Ọmụgwọ I was not invited for. … I, therefore, believe Ọmụgwọ does preserve health and well-being for both mother and child. (Mrs. Onyi, 61 years old)

#### Practice continuity irrespective

Again, it was noted that participants do not perceive Ọmụgwọ as labor needing any form of remuneration from their care recipient. Hence, they are happy to continue attending Ọmụgwọ practice with or without payment and amid financial loss.

... I have attended many Ọmụgwọ and I have no reservations about the practice. I have never looked at it from the angle of payment and do not encourage anyone to look at it from that perspective… Ọmụgwọ is an age-long practice which should continue exactly the way it is. (Mrs. Uwaoma, 71 years old)

Other participants supported this claim as captured further by the narrative of a participant:

We don’t need to be paid to attend Ọmụgwọ and I believe the practice should not be seen as labor that needs salary. This is not debatable in Igboland (Southeast, Nigeria) and I wonder where such an idea emanated. …hmmm, pay me a salary to attend to my daughter’s Ọmụgwọ. That’s unheard of please … yes it should continue. (Mrs. Chichi, 64 years old)

Some of the participants confirmed that even though they do not call for payment, the Ọmụgwọ practice makes them suffer financial loss, especially for those who are economically engaged:

I would give anything to go for my grandchild’s Ọmụgwọ, however, I must confess that it usually takes a toll on my pocket (financial loss). For instance, I sometimes transport myself to and fro, and I buy a lot of stuff in preparation for Ọmụgwọ. (Mrs. Onyi, 61 years old)

…the last time I went to Ọmụgwọ, my shop was locked for three months. … no income for 3 months yet I was spending from my savings. Still, this cannot deter me from attending the next Ọmụgwọ. (Mrs. Maggi, 60 years old)

Again, participants continued to make comments to establish their financial loss and economic disengagement, disapproval of salaries for Ọmụgwọ, and the need for the practice to continue irrespective of the economic disadvantage.

### Theme 2: Cultural Influences

Study analysis shows that culture shapes the perceptions and experiences of participants regarding Ọmụgwọ practice. According to them, their culture is deeply rooted in the concept of reciprocity, and parental responsibilities highlighting the intergenerational support embedded in the African culture as participants pay forward the care they received. The fulfillment of these responsibilities brings social praise and increased ego for grandmothers.

It is cultural for me and other Igbo grandmothers to attend Ọmụgwọ and provide the needed support to our children. It’s an age-long tradition passed down from mothers to daughters. …for instance, my great-grandmother attended Ọmụgwọ of my mother because she (grandmother) received the same support and care from her mother during her own Ọmụgwọ. (Mrs. Akudo, 60 years old)

#### Care reciprocity and responsibility

The African culture of family care influences the views and experiences of participants regarding Ọmụgwọ. Participants believed that it is customary to care for their children, in return for the care they received from their parents and to be responsible for their children in exceptional situations in adult age. The views of the participants on depicting reciprocity of care relationships in family caregiving as expressed are illustrated with the following quotes:

Yes, my mother came for my Ọmụgwọ and cared for me and my baby. My mother was always there each time I put to bed (have a baby). I, therefore, felt I owe the same care to my children; hence I have always attended all my daughters’ Ọmụgwọ without expecting anything in return… In return, my mother has attended two of my Ọmụgwọ because she felt obligated to do the same as she received. I also felt obligated to return the same care when I traveled to Lagos and abroad to provide care for my two daughters who were nursing mothers. (Mrs. Okeke, 65 years old)

If I don’t take care of my children during Ọmụgwọ, who will? No one will take up our responsibilities, especially when it is unpaid. While our adult children care for themselves, we still owe it as a duty to provide care for them when they are in vulnerable situations such as being hospitalized or during Ọmụgwọ. (Mrs. Ngo, 60 years old)

#### Social status and self-esteem

Increased social status and ego were noted among participants for having attended Ọmụgwọ. They reported that participation in Ọmụgwọ accords them more respect among their peers and in social gatherings and the reverse is shame and stigma. Two participants said:

…in our culture, there are many metrics for measuring the social status of women. Among these metrics include childbearing, becoming a grandmother, and attending Ọmụgwọ of your grandchild. Sometimes, those who haven’t experienced the Ọmụgwọ practice are the subject of mockery. (Mrs. Chichi, 64 years old)

…It was shameful to me each time my mates congratulate me on my daughter’s delivery and probed to know why I haven’t gone for Ọmụgwọ. I became the talk of the town. This made me cry out to my daughter until I was finally invited. Upon return from the Ọmụgwọ, I became a ‘celebrity’ in the village and regained my respect and rights. I am always quick to tell people that I just returned from Ọmụgwọ. (Mrs. Chukwu, 61 years old)

Other participants continued to cite examples of how culture influences their perception and experiences of Ọmụgwọ.

### Theme 3: Indirect Care Reward/Benefits for Grandmothers

Analysis of the data further shows that participants do not feel they are losing out entirely by engaging in Ọmụgwọ as most (*n* = 15) reported other different types of rewards or benefits they get from Ọmụgwọ. Participants reported both material and immaterial gains such as decreased loneliness and tourism among other material compensations.

#### Decreased loneliness

Many of the participants (*n* = 12) believed Ọmụgwọ enables them to stay closer to their children and grandchildren and as such, decreases their feeling of loneliness: “I’m always alone and lonely in the village. … Ọmụgwọ is most cherished because it helps me navigate the feeling of loneliness. Seeing and speaking with my children often gives me joy” (Mrs. Akudo, 60 years old). Some of the participants (*n* = 5) also agreed that traveling to the city for Ọmụgwọ creates an opportunity for frequent contact with their other children, relatives, and friends:

… friends, families, and relatives from near and far always come to visit the newborn. My other children who reside in the city also visit often because it is a time for celebration. Because the house is always full of visitors, I didn’t get to feel as lonely as I felt back in the village. (Mrs. Ihuoma, 61 years old)

Another respondent had this to say:

… Ọmụgwọ is usually my only opportunity to freely travel to my children’s house to spend one or two months. I cherish those moments because it helps me feel less lonely. (Mrs. Chinenye, 64 years old)

#### Tangible gifts

Although participants do not see the need to receive payments for Ọmụgwọ, they, however, often get material compensation. Most of the participants (*n* = 14) reported getting gift items including jewelry, clothes, and often cash and foodstuffs upon completion of their stay. Some of the participants recounted their experiences thus:

During Ọmụgwọ, my in-law usually buys me things than usual so I can enjoy my stay at their place. Sometimes my in-law takes me shopping and ensures I get everything I request. Yes ... Ọmụgwọ is a tedious process full of sleepless nights, but I enjoyed myself as I was given a 5-star treatment. My daughter and her husband also gave me lots of gifts, food items, and money. (Mrs. Ugodi, 60 years old)

Aside from the other gift items my in-law gave me upon completion of my last Ọmụgwọ, he also gave me enough money to open a provision store in appreciation for the care I provided. (Mrs. Victoria, 69 years old)

Some of the participants reported receiving little to no gifts during their last Ọmụgwọ and attributed it to the socioeconomic status of their children who were struggling at the time.

#### Tourism

The analysis further shows that participants believe Ọmụgwọ provides them with tourism opportunities. All the participants have had the opportunity to travel and visit new places. Although most (*n* = 14) have traveled locally, others (*n* = 3) have traveled internationally for Ọmụgwọ. The narratives of three of our participants capturing views of others, sampled thus: “I was happy to visit Lagos about three years ago. …What else would have taken me to Lagos if not Ọmụgwọ. I always see Lagos on television, but Ọmụgwọ made it possible for me to travel there and behold the beauty of the city” (Mrs. Amaka, 63 years old).

I just came back from the United States of America where I went for Ọmụgwọ. My in-law sponsored the trip, and it was indeed an opportunity of a lifetime for me…If not for Ọmụgwọ. (Mrs. Joy, 60 years old)

## Discussion

The WHO has recommended the need for postpartum care for nursing mothers and their newborns (WHO, 2015). Hiring professionals such as retired nurses and midwives to render postpartum care is an alternative greatly explored in western countries (Vermeulen et al., 2019; Williams & Cooper, 1993). However, the same cannot be said of caregiving in Sub-Saharan Africa, where the option of hiring professionals is greatly overlooked and substituted for family caregiving (Iwuagwu & Kalu, 2021; Iwuagwu, Lai, et al., 2022; Mom, 2022). The current study set out to investigate the perceptions of grandmothers about Ọmụgwọ, with the aim of understanding and learning from their experiences. This empirical research is the first of its type in Nigeria, and it reveals some key conclusions about their perceptions and experiences regarding the place of remuneration for the time spent in Ọmụgwọ. Despite its unpaid nature, participants in this study unanimously perceived Ọmụgwọ positively and expressed their desire to continue the practice.

According to WHO (2022a) report, “the need for quality maternity and newborn care does not stop once a baby is born because post-birth can cause unprecedented stress and anxiety. Parents, therefore, need adequate health care and support systems” (p. 1). In agreement with the above statement, the findings of this study show that respondents hold views supporting the continuity of Ọmụgwọ practice because, from their lived experiences, Ọmụgwọ has helped reduce stress for nursing mothers and further reduced infant and maternal deaths. For instance, participants reported providing experienced care for the nursing mother and newborn while helping the nursing mother to achieve less physical and psychological distress accompanying postpartum care. Further review of a WHO document aligns with the findings of this research. For instance, the document shows the importance of having a caregiver who is experienced to recognize issues that call for additional care and provide guidance on when to take a newborn to a medical facility. They could also provide counseling on how to identify danger indicators, comprehend the care that both the mother and the baby require, and know where to find such services when needed (WHO, 2022b). Although not among older grandmothers, other studies have reported similar findings about the benefits of having an experienced carer, such as reduced stress and mortality associated with care provision for nursing mothers and their babies. For instance, in Uganda, Beinempaka et al. (2015) argued that after-birth care (Ọmụgwọ) is beneficial in many ways including the enhancement of maternal rest and experienced care for the newborn. Tiruneh and colleagues systematically mapped the literature of randomized trials and quasi-experimental studies, while using a random-effect meta-analysis model to test the effectiveness of home-based postpartum care (2019). Their result shows that home-based postpartum care reduces neonatal mortality (Tiruneh et al., 2019). Gray literature has supported this finding also. For instance, Dimeji-Ajayi (2018) was quoted as saying “Ọmụgwọ, is necessary so that the new mother can rest well to regain her strength” (p. 5). Findings further show that participants agreed to continue attending Ọmụgwọ and provide unpaid care, partly because of the perceived and experienced benefits of Ọmụgwọ on nursing mothers and newborns.

Culture, perception, and behavior are inextricably linked. Literature has shown that through the lens of culture, people’s actions and perceptions can be determined. For instance, McCleary and Blain’s (2013) review study shows the influence of filial piety and familism as cultural values influencing caregiving practice. The findings of the present study show that the African culture of care reciprocity influences older grandmother’s views and experiences of Ọmụgwọ. Although the pressure cuts across the locality divide, those in rural areas are more susceptible to such cultural pressures as deduced from the data analysis. This is not surprising because, in Nigeria, the rural population is more cultural as the environment is more homogenous in culture compared to the heterogeneity of urban areas (Nwauzor, 2017). Although not among older grandmothers, Dike (2019) found that culture influences first-generation Nigerian women’s birth experiences, and Lawal and colleagues concluded that Ọmụgwọ is a replication of old African tradition (2022). Other caregiving literature has documented similar reports of care reciprocity as an African cultural belief influencing their views and experiences (Akinrolie et al., 2020; Iwuagwu & Kalu, 2021; Iwuagwu, Lai, et al., 2022; Ojembe & Kalu, 2018). Globally, parents have the duty to protect and care for their children until they reach adult age (New South Wales Government, 2020). However, in Africa, parents continue to assume some care responsibilities for their mature children especially those in vulnerable situations including nursing mothers. This present study shows that older grandmothers believed that attending Ọmụgwọ and caring for their mature daughters was their responsibility. Older grandmothers also reported increased social status upon return from Ọmụgwọ as they were praised. These are inextricably linked to cultural reverence for Ọmụgwọ in Nigeria and Africa.

Offering services often come with a direct reward of receiving a paycheck. However, there is another aspect of remuneration that is crucial for service delivery known as indirect compensation (Verlinden, 2022). Indirect compensation includes other nonmonetary benefits gotten from completing tasks (Boyce & Geller, 2001). This study found that although older grandmothers in Nigeria are not compensated financially, they are happy with the indirect rewards they get from Ọmụgwọ such as decreased loneliness, material compensation (tangible gifts), and the opportunity for local and international tourism. Scholars have argued that although these rewards may be indirect, they often have monetary value (Boyce & Geller, 2001; Verlinden, 2022). Because Ojembe and Kalu (2018) argued that older adults’ loneliness has increased because of the rural–urban migration of their children, the National Institute on Aging (2022) avers that people including older grandmothers, who participate in enjoyable, fulfilling activities with others have a feeling of purpose, are less lonely and typically live longer. Findings support this argument by reporting that older grandmothers who travel for Ọmụgwọ in cities or other countries feel less lonely as they are closer to their children and grandchildren, and Ọmụgwọ is enjoyable and relished. Other studies have also reported the interconnection between loneliness and proximity to children and grandchildren (Iwuagwu & Kalu, 2021; Ojembe & Kalu, 2018; Ojembe et al., 2021, 2022).

Findings also show other means of compensation including material goods. Ujumadu (2018) in an opinion piece confirmed this finding by asserting that it is cultural in Southeast Nigeria for mothers-in-law to be given gifts such as food items, clothing, shoes, etc. as an appreciation for time spent during Ọmụgwọ. These gifts, however, differ according to the socioeconomic status of the son-in-law, and it is not statutory. The findings of the present study also show that Ọmụgwọ is an opportunity for tourism. Although there is a paucity of literature to substantiate this finding, Dike (2019) conducted a qualitative inquiry into the birth experiences of first-generation Nigerian mothers residing in London. Although not among older grandmothers, the findings of Dike confirmed claims of the present research finding about Ọmụgwọ facilitating both local and international travels for grandmothers as an indirect reward (Dike, 2019). Findings show that those who are economically engaged reported more loss and less compensation from Ọmụgwọ compared to their mates who are unemployed or retired. This, therefore, aligns with the literature about the intersectionality of age, gender (being old and female), and Ọmụgwọ experience having a significant impact on the income of women, throwing them at financial risk as they age without benefits. Also similar to the findings of this study, Feldman and Radermacher’s (2016) study concludes that women in Sub-Saharan Africa face financial risk due to their care responsibilities and frequent disengagement from the labor market.

### Study Implication

The literature has shown that older adults generally encounter issues of loneliness, social isolation, poor finances, deteriorating health, etc., and are at risk of successful aging (Ebimgbo et al., 2019; Ekoh et al., 2022; Iwuagwu & Kalu, 2021). The implication of Ọmụgwọ on older grandmothers is enormous, especially in Sub-Saharan Africa, where the practice is cultural and revered. Gerontological social workers among other clinicians should leverage these experiences of Ọmụgwọ to improve the health and mental well-being of these populations in the region. For instance, by building on their cultural views of Ọmụgwọ, gerontological social workers could help facilitate invites and travels for Ọmụgwọ as this could potentially reduce social loneliness among their patients. Although older grandmothers are happy to engage in Ọmụgwọ without any form of payment, there is a dire need for gerontological social workers to champion aging policies aimed at providing statutory social incentives from the government purse to older grandmothers who are providing postpartum care. For instance, the government should provide transport fare for older adults to provide Ọmụgwọ for their children and grandchildren. Further, if the government in Nigeria and Sub-Saharan Africa institutionalized Ọmụgwọ practice and provides some support for families and older grandmothers who attend Ọmụgwọ, it would help remove pressure from nursing parents who are struggling economically, and consequently unable to invite their parents to Ọmụgwọ. Lastly, this policy measure will enable older grandmothers, especially those who are economically engaged to feel less pressured by economic disadvantage and more relaxed during Ọmụgwọ. This will in turn promote quality and longer-term Ọmụgwọ.

#### Strengths and limitations

Although this study is the first to empirically investigate the experiences of older grandmothers about Ọmụgwọ in Nigeria and has further strengths, it is not without some limitations. The study’s strength centers on the application of strategies to ensure rigor such as participant-member checking, reflexivity throughout the research process, the use of detailed and thick descriptions of the study process for other researchers to replicate, the use of an expert translator, etc. However, the findings of this research reflect the views of only 17 older grandmothers which is a small subset in Southeast Nigeria. Caution should therefore be applied in generalizing these findings as results may be different in a broader population and region. Future studies should build on this and cover other regions to compare findings. The use of one coder could also be a limitation of this study. However, the author shared and received feedback from some groups of anonymous researchers to minimize issues in the coding process. Although the interview guide which was not translated into the Igbo language may also be a limitation, the interview was conducted by the researcher who is fluent in both English and Igbo language and further, employed different strategies including expert Igbo translators of interviews in English, and participant member check at study end to confirm findings.

## Conclusion

Ọmụgwọ practice is important and perceived positively by older grandmothers in Nigeria because of its cultural implications. Although older grandmothers do not seek remuneration for the practice, they often received some other forms of compensation, which however do not equate to the care they provided. Given the importance of Ọmụgwọ to the health and well-being of the family, it is recommended that the government steps in to adequately compensate older grandmothers who attend Ọmụgwọ.

## Supplementary Material

igad069_suppl_Supplementary_Material
