# Supplementary material for: “Ọmụgwọ” As Unpaid Labor? The Perceptions of Postpartum Caregiving Among Older Grandmothers in Southeast Nigeria
Source: Innov Aging. 2023 Jul 13;8(4):igad069. doi: 10.1093/geroni/igad069 (PMC10993719; doi:10.1093/geroni/igad069)
Supplement: igad069_suppl_Supplementary_Material [file igad069_suppl_supplementary_material.docx]

**Online Supplementary Material**

**Example Coding Process**

| **Units of Analysis** | **Codes** | **Categories** | **Subthemes** | **Themes** |
| --- | --- | --- | --- | --- |
| Yes, it was my mother who came for my Ọmụgwọ and cared for me and my baby. My mother was always there each time I put to bed. I, therefore, felt I owe the same care to my children; hence I have always attended to all my daughters’ Ọmụgwọ without expecting anything in return… (Mrs. Akudo, 60 years old)*.* | Cultural reciprocity and caregiving | Reciprocal care | Care reciprocity and responsibility | **Cultural Influences** |
| "It is cultural for me and other Igbo grandmothers to attend Ọmụgwọ and provide the needed support to our children. It's an age-long tradition passed down from mothers to daughters. …for instance, my great grandmother attended Ọmụgwọ of my mother because she (grandmother) received the same support and care from her mother during her own Ọmụgwọ. In return, my mother has attended two of my Ọmụgwọ because she felt obligated to do the same as she received. I also felt obligated to return the same care when I traveled to Lagos and abroad to provide care for my two daughters who were nursing mothers (Mrs. Okeke, 65 years old). | Age long tradition |  |  |  |
| If I don't take care of my children during Ọmụgwọ, who will? No one will take up our responsibilities, especially when it is unpaid. While our adult children care for themselves, we still owe it as a duty to provide care for them when they are in vulnerable situations such as being hospitalized or during Ọmụgwọ (Mrs. Ngo, 60 years old). | Cultural beliefs | Sense of responsibility |  |  |
| …in our culture, there are many metrics for measuring the social status of women. Among these metrics include childbearing, becoming a grandmother, and attending Ọmụgwọ of your grandchild. Sometimes, those who haven't experienced the Ọmụgwọ practice are subject of mockery (Mrs. Chichi, 64 years old). | Culture | Stigmatization from cultural practices | Social status and self-esteem |  |
| …It was shameful to me each time my mates congratulate me on my daughter’s child delivery and probed to know why I haven't gone for Ọmụgwọ. I became the talk of the town. This made me cry out to my daughter until I was finally invited. Upon return from the Ọmụgwọ, I somewhat became a celebrity in the village, and I regained my respect and rights. I am always quick to tell people that I just returned from Ọmụgwọ (Mrs. Chukwu, 61 years old). | Esteem issues | Social praise upon return from Ọmụgwọ |  |  |
